# Waist Circumference and BMI Are Strongly Correlated with MRI-Derived Fat Compartments in Young Adults

**DOI:** 10.3390/life11070643

**Published:** 2021-07-01

**Authors:** Duanghathai Pasanta, Khin Thandar Htun, Jie Pan, Montree Tungjai, Siriprapa Kaewjaeng, Sirirat Chancharunee, Singkome Tima, Hong Joo Kim, Jakrapong Kæwkhao, Suchart Kothan

**Affiliations:** 1 Center of Radiation Research and Medical Imaging, Department of Radiologic Technology, Faculty of Associated Medical Sciences, Chiang Mai University, Chiang Mai 50200, Thailand; duanghathai.pas@cmu.ac.th (D.P.); ktdhtun28@gmail.com (K.T.H.); jiepan@sdnu.edu.cn (J.P.); mtungjai@gmail.com (M.T.); siriprapa.k@cmu.ac.th (S.K.); 2 Shandong Provincial Key Laboratory of Animal Resistant Biology, College of Life Sciences, Shandong Normal University, Jinan 250014, China; 3 Department of Chemistry, Faculty of Science, Chiang Mai University, Chiang Mai 50200, Thailand; c.sirirat@gmail.com; 4 Department of Medical Technology, Faculty of Associated Medical Sciences, Chiang Mai University, Chiang Mai 50200, Thailand; singkome.tima@cmu.ac.th; 5 Department of Physics, Kyungpook National University, Daegu 41566, Korea; hongjoo@knu.ac.kr; 6 Center of Excellence in Glass Technology and Materials Science (CEGM), Nakhon Pathom Rajabhat University, Nakhon Pathom 73000, Thailand; jakrapong@webmail.npru.ac.th

**Keywords:** magnetic resonance spectroscopy, abdominal fat, visceral fat, subcutaneous fat, young adult, body-mass index, waist circumference, waist-to-hip ratio

## Abstract

Young adulthood is increasingly considered as a vulnerable age group for significant weight gain, and it is apparent that there is an increasing number of new cases of metabolic syndrome developing among this population. This study included 60 young adult volunteers (18–26 years old). All participants obtained a calculated total abdominal fat percentage, subcutaneous fat percentage, and visceral fat percentage using a semiautomatic segmentation technique from T1-weighted magnetic resonance imaging (MRI) images of the abdomen. The results show strongest correlation between abdominal fat and BMI (r = 0.824) followed by subcutaneous fat (r = 0.768), and visceral fat (r = 0.633) respectively, (*p* < 0.001 for all, after having been adjusted for age and gender). Among anthropometric measurements, waist circumference showed strong correlation with all fat compartments (r = 0.737 for abdominal, r = 0.707 for subcutaneous fat, and r = 0.512 for visceral fat; *p* < 0.001 for all). The results obtained from examining the blood revealed that there was a moderate positive correlation relationship between all fat compartments with triglyceride, high-density lipoprotein, and fasting glucose levels (*p* < 0.05 for all). This study suggests that both BMI and waist circumference could be used to assess the fat compartments and treatment targets to reduce the risk of metabolic disorders and health risks in the young adult population.

## 1. Introduction

Young adulthood is usually described as the period between 18–30 years of age [1] and is the transition phase from adolescence into adulthood. There is a growing body of literature that recognizes this age group as a vulnerable time for developing unhealthy lifestyles and poor dietary habits and is also at risk of significant weight gain [2]. These negative habits have been associated with the alteration of the environment, academic stress, responsibility, and independent lifestyles during this transition stage [3]. There are reports that poor diet behaviors and weight gained during earlier life stages are likely to last into adulthood, exposing these people to a higher risk of metabolic syndrome [4,5].

A metabolic syndrome is a group of risk factors that increase the likelihood of developing cardiovascular disease (CVD), stroke, and type 2 diabetes [6,7]. These risk factors include dyslipidemia, dysglycaemia, central obesity, hypertension, and insulin resistance [7]. Metabolic syndrome was present in 4.8–7% of healthy young adults (18–30 years old), and abdominal fat is the third most prevalent factor in young adults with metabolic syndrome [8,9]. Intraperitoneal fat or visceral fat is reported to be one of the key factors underlying metabolic syndrome. These factors have also been found to be associated with CVD and certain types of cancers and contribute to overall morbidity and mortality [10,11]. Computed tomography (CT) and dual X-ray absorptiometry (DXA) are well-established techniques used in clinical and scientific research to evaluate abdominal adipose tissue compartments [12,13]. These techniques use radiation in measurement and therefore are unsuitable for frequent assessments and longitudinal studies. To overcome these disadvantages, a safer and more effective technique needs to be established. Magnetic resonance imaging (MRI) holds the advantage of being a non-invasive and non-radiation technique with excellent soft tissue contrast for evaluating abdominal fat distribution. For these reasons, MRI has become the preferred method for the quantification of visceral fat and subcutaneous fat in both adults and adolescents [14,15]. MRI techniques such as T1-weighted imaging, magnetic resonance spectroscopy (MRS), and fat-water imaging sequence (chemical shift-based) have been used extensively to assess body fat in both research and clinical areas [16,17,18,19]. However, there has been little quantitative analysis data obtained concerning the use of 1.5 T MRI techniques for fat compartments in young adults, despite the importance of an association between abdominal fat and associated health risks in this age group.

The purpose of this study is to analyze the total abdominal fat, subcutaneous fat, and abdominal visceral adipose tissue compartments of young participants using 1.5 T MRI images, combined with anthropometric parameters and laboratory measurements to determine the risk factors for metabolic syndrome and to comprehensively assess the association between abdominal adipose tissue and metabolic syndrome in this age group.

## 2. Materials and Methods

### 2.1. Participants

The participants included 60 young adults aged 18–26 years old (27 male and 33 females). The exclusion criteria included chronic disease; body mass index (BMI) in the obese range (≥30 kg/m^2^) or underweight range (<18.5 kg/m^2^); under-medication that affects metabolism (i.e., insulin, blood sugar, or lipid level); any contraindication for MRI or low MR abdominal image resolution. All of the female participants stated that they were not pregnant and were not using any form of contraception.

### 2.2. Ethical Considerations

All participants signed written informed consent obtained after fully understanding the nature of the study during the recruitment period. The study was conducted in accordance with the Declaration of Helsinki, and the protocol was approved by the Ethics Committee of the Faculty of Associated Medical Sciences, Chiang Mai University, Chiang Mai, Thailand (AMSEC-61EX-016).

### 2.3. Anthropometry

All participants were measured for anthropometric measurements. Each one had a blood examination, and they all underwent MRI for MR imaging of the abdomen on the same day. Weight and height were measured twice by the same examiner throughout the study. BMI was calculated by dividing the body weight (kg) by height in meters and was expressed as kg/m^2^. Hip circumference (HC) and waist circumference (WC) were measured twice in a standing position with a non-elastic tape measure, while participants were instructed to breathe out mildly with measurements being recorded to the nearest 0.5 cm. WC was measured at the midpoint between the top of the iliac crest and the lowest coastal rib. HC was measured around the pelvis at the largest part of the buttocks. The waist-to-hip ratio (W/H ratio) was then calculated.

### 2.4. Biochemical Analysis of Blood

All laboratory examinations were conducted by The Associated Medical Science Clinical Service Center, Chiang Mai University and were biochemically analyzed using a fully automated analyzer (Architect ci8200, Abbott Diagnostic, Santa Clara, CA, USA). Intravenous blood samples were drawn after an overnight fast of 10–12 h. Blood lipids (Cholesterol (Cho), high-density lipoproteins (HDLs), very-low-density lipoproteins (VLDLs), triglycerides (TG), fasting glucose (FG), and glycated hemoglobin (HbA1c) were measured. LDL levels in this study were calculated from the novel method LDL using the estimation factor [20,21,22]. In this study, dyslipidemia was defined as Cho ≥ 200 mg/dL, Tri ≥ 150 mg/dL, LDL ≥ 130 mg/dL, and HDL ≤ 40 mg/dL [23]. The normal FG range was defined to be between 70–100 mg/dL, and normal HbA1c levels should be less than 6% [24].

### 2.5. MRI Images Acquisition and Image Processing

A 1.5 Tesla Philips Ingenia MR imaging machine (Philips Healthcare, Amsterdam, The Netherlands) equipped with a SENSE cardiac coil was used. The T1-weighted axial images of the abdomen were obtained using the following parameters: Repetition time (TR) = 10 ms, echo time (TE) = 5 ms, slice thickness = 7.0 mm, gap = 1.0 mm, field of view (FOV) = 375 mm, and a 7 mm thickness slice at the level of L3–L4 discs was selected for the quantification of fat, as it is free from the influence of liver or adipose tissue from the buttocks.

**Figure 1:**
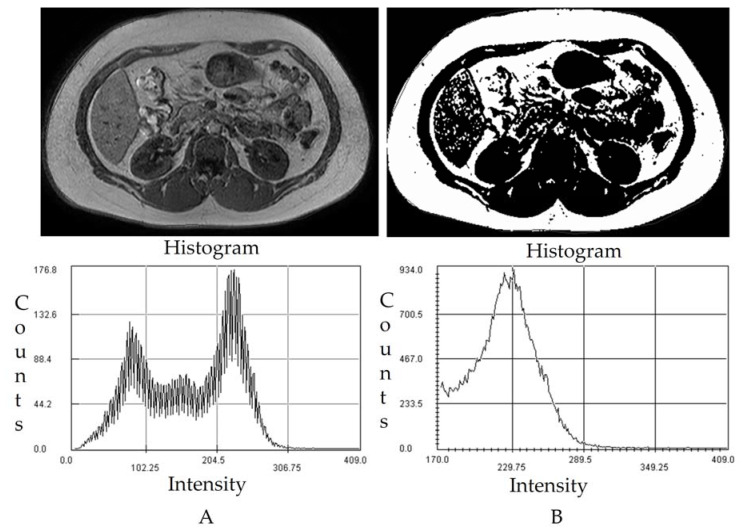
Magnetic Resonance Imaging of the Abdomen using a semiautomatic segmentation technique to estimate abdominal compartments using a histogram. (**A**) The image shows intra-abdominal (visceral) and subcutaneous fat. T1 weighted DICOM MRI image from L3–L4. (**B**) Binary Image obtained from semiautomatic segmentation technique for abdominal fat measurement.

The T1-weighted images were then analyzed using Medical Image Processing, Analysis, and Visualization (MIPAV, National Institutes of Health, Bethesda, MD, USA) software package using a semiautomatic segmentation technique that converts grayscale pixels into binary black and white images based on the signal intensity-based histogram-thresholding method [16]. The areas with high signal intensity or which appeared to be brighter represented abdominal adipose tissue and were set as the threshold to exclude the non-relevant organs and tissue in the image [25]. The pixel values that appeared as black in the binary image represent abdominal adipose tissue, while the white pixel represents soft tissue such as muscle, blood vessels, and bony structures (Figure 1). Areas of visceral and subcutaneous fat were determined from the image through the manually drawn region of interest (ROI) along the abdominal wall to separate intra and extra-abdominal boundary with the manual exclusion of the non-adipose tissue area [16]. Afterwards, the total abdominal fat percentage (AbdFat%), subcutaneous fat percentage (ScFat%), and visceral fat percentages (VisFat%) were calculated.

### 2.6. Statistical Analysis

Data are expressed as mean ± standard deviation (SD) unless stated otherwise. The Kolmogorov–Smirnov test and the Shapiro–Wilk test were performed to determine data normality. The unpaired samples *t*-test was used to compare various parameters between males and females. Partial correlation analysis was used to verify abdominal fat compartments and models from blood and anthropometric measurements. Multiple stepwise linear regression analysis was used to verify the relationships between abdominal fat compartments and models from blood and anthropometric measures. Results with a *p*-value < 0.05 were considered statistically significant. Statistical analysis was performed using R version R-4.0.3.

## 3. Results

### 3.1. Anthropometric

The characteristics of the participants in this study are shown in Table 1. A total of 60 participants and an average BMI of 25.96 ± 5.48 kg/m^2^, WC 87.29 ± 15.57 cm, HC 99.91 ± 12.39 cm, and W/H ratio 0.87 ± 0.08 were included in this study. From Table 1, results show statistically significant lower BMI, WC, W/H ratio in females compared to males. While HC was not statistically different between males and females.

### 3.2. Abdominal Fat Compartments and Laboratory Characteristics

**Figure 2:**
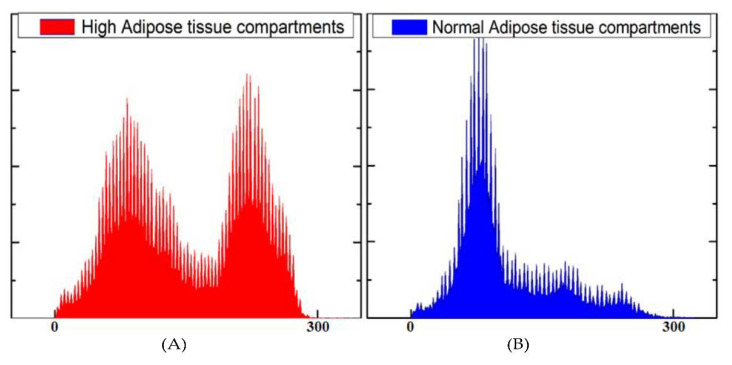
Histograms of pixel intensities showed two peaks of gray values to differentiate adipose tissue. (**A**) Representative signal histograms obtained from T1-weighted abdominal MR images with high overall adipose tissue compartments. (**B**) Representative signal histograms were obtained from T1-weighted abdominal MR images with typical amounts of adipose tissue compartments.

Using MR images, the axial images at the L3–L4 level were analyzed by semiautomatic signal intensity based on MIPAV free software. Histograms of pixel intensities showed two peaks of gray values to differentiate adipose tissue from non-adipose tissue. From the histogram, the difference between normal and high adipose tissue compartments can be seen (Figure 2).

The average fat content percent for AbdFat%, ScFat%, and VisFat% were 33.91 ± 12.25%, 22.48 ± 9.63%, and 10.63 ± 4.95% for males, 27.99 ± 12.72%, 21.80 ± 9.99%, and 5.42 ± 3.41% for females, respectively. The results show statistically significant differences in VisFat%, Tri, and HDL between males and females, but there were no significant differences in AbdFat, ScFat, FG, Cho, LDL, and HbA1c.

**Figure 3:**
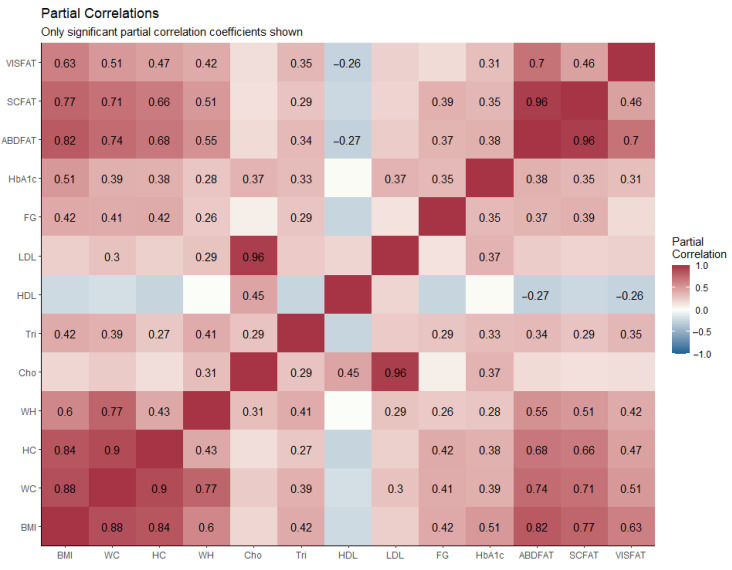
Partial correlation matrix showing the correlations between demographics, abdominal fat compartments, anthropometric, and laboratory characteristics. Only significant partial correlation coefficients are shown, Results with a *p*-value < 0.05 were considered statistically significant. BMI = body mass index, Cho = cholesterol, FG = fasting glucose, HbA1c = glycated hemoglobin, HC = hip circumference, HDL = high-density lipoprotein, LDL = low-density lipoprotein, Tri = triglyceride, W/H = waist hip ratio, WC = waist circumference.

**Figure 4:**
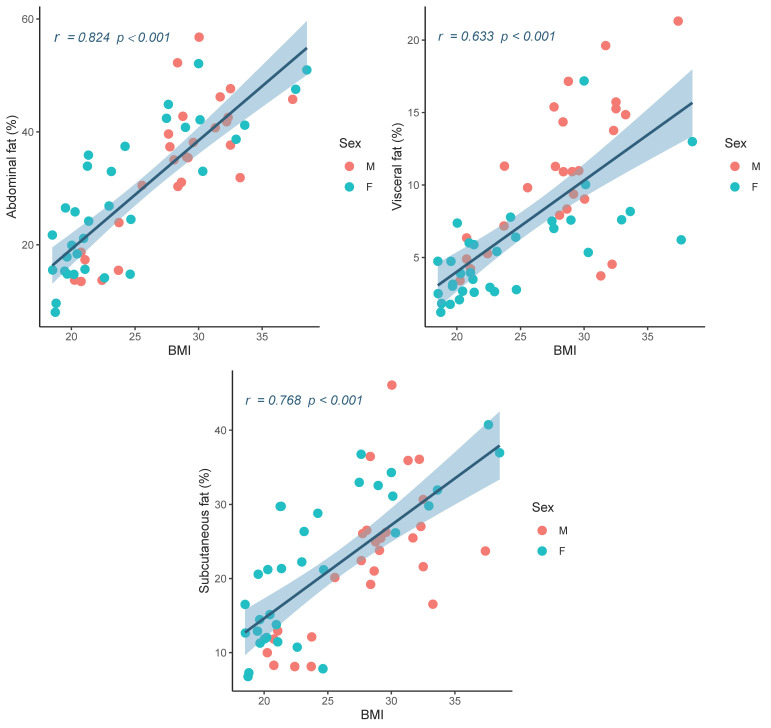
Scatter plots between percentages of abdominal fat, visceral fat and subcutaneous fat with BMI show positive correlations.

The correlations between lipid compartments, anthropometric, and laboratory measurements are presented in Table 2 and Figure 3. AbdFat%, ScFat%, and VisFat% were highly correlated with BMI (*p* < 0.001 for all). WC also showed a high correlation with AbdFat%, ScFat%, and VisFat%, while HC and W/H are moderately correlated (*p* < 0.001 for all). The results obtained from the blood laboratory examination also presented the relationship between the elevation of AbdFat%, ScFat%, and VisFat%, with Tri. There was a mild association between increased LDL and elevated AbdFat% and VisFat%, but not ScFat%. However, there was a mild negative association between HDL and AbdFat% (*p* = 0.040) and VisFat% (*p* = 0.048), but there was no significant correlation with ScFat%. FG and HbA1c also showed a mild, but statistically significant, association with AbdFat% (r = 0.381, *p* = 0.003), ScFat% (r = 0.351, *p* = 0.007), and VisFat% (r = 0.315, *p* = 0.016). The scatter plot of AbdFat%, ScFat% and VisFat% with BMI is shown in Figure 4. The results show correlations were in the same trend for both males and females, revealing that the relationships between BMI and fat compartments hold true regardless of gender.

## 4. Discussion

The accumulation of abdominal adipose tissue fat deposition is believed to be associated with a higher risk of developing CVD and metabolic syndrome [26,27]. These relationships between the abdominal fat compartments could be used as early detection tools to assess the risk of metabolic syndrome and its related diseases [26]. This study has shown that MR images allow for simple, non-invasive, and non-radiation examinations and is an efficient method for the semi-automatic identification and quantification of fat compartments, including abdominal, subcutaneous, and visceral fat in the young adult population.

The results of the current study showed that AbdFat%, ScFat%, and VisFat%, measured using MRI technique, were significantly and strongly correlated with BMI and WC. While HC and the W/H ratio showed a moderate and significant association with abdominal fat compartments. This study supports the evidence obtained from previous reports that WC and BMI can be incorporated as a non-invasive screening tool to predict the total amount of subcutaneous and abdominal fat in order to identify the increased risk of metabolic syndrome [26,28]. This work also further supports the idea from other studies in this area that link BMI and WC as a more accurate tool to assess the visceral adiposity in young adults [29,30]. However, the outcome of this current study is contrary to the previous findings that BMI did not correlate with the amount of abdominal adiposity [10].

It is interesting to note that elevated WC is associated with an increased risk of developing a cardiovascular disease, which supports the inclusion of WC measurements during the routine medical assessment of adolescents [31,32,33,34]. This present study showed that Tri, FG, and HbA1c were weakly associated with AbdFat%, ScFat%, and VisFat%. Our study also demonstrated that subcutaneous adipose tissue was not associated with changes in either the LDL or HDL lipoprotein profiles. A significantly weak positive correlation of LDL and VisFat% was observed. There is also a significant but weak negative correlation between HDL and abdominal fat percentage, except for ScFat%. However, no correlation between Cho with abdominal adipose tissue depots was found.

This result may be explained by the fact that visceral fat has greater lipolytic activity compared to subcutaneous fat and may reduce hepatic insulin sensitivity, and it also favors hepatic fat accumulation probably due to high free fatty acid (FFA) flux to the liver and thereby promotes an atherogenic lipid profile [35]. This result is in agreement with the former findings of visceral adipose tissue, which were positively related to LDL and was negatively associated with HDL [36,37,38], confirming the association between the visceral fat and its impact on atherogenic lipoprotein profile. This also suggests that it has a less important role than subcutaneous fat on blood lipoprotein profiles [39]. Likewise, the positive correlation of Tri in the current study supports the evidence from previous observations that hypertriglyceridemia constituted the major lipid profile alterations, especially among the viscerally obese that also showed the highest correlation among abdominal fat depots [27]. Consistent with the literature, this research found that high visceral adipose tissue is associated with the risks factors of metabolic syndrome [40,41], such as increasing Tri and an impaired FG and HbA1c, along with a trend towards decreased HDL levels.

These results support several studies that proposed that chemical shift-based MRI with complete fat-water tissue separation is an effective and accurate cross-sectional technique for both the identification and quantification of subcutaneous and visceral fat [16,38,42]. This technique is suitable for longitudinal follow-ups in young adults when compared to the gold standard for abdominal fat measurements, such as CT scans, and is able to assess the abdominal compartment without the risk of unnecessary exposure to radiation. MRI-derived fat compartments are highly sensitive, reliable, and have been reported to be highly accurate [43,44]. MRI supports a greater understanding of the role of body fat accumulation in the physiology and pathophysiology of obesity, aging, and metabolic diseases.

### Limitations and Directions for Future Research

The scope of this study was limited in terms of age as only young adults between 18 and 26 years of age were included. Thus far, this work has focused on the ability to identify and quantify abdominal fat depots from MR images. Another imperfection was the number of participants, such that an additional study using a larger group of participants would need to be undertaken to validate these findings. Additionally, we did not investigate the effect of gender in great detail, and future studies should further investigate this topic. While the current study is based on a small sample of participants, the findings have confirmed that simple anthropometric measurements, such as BMI, WC, and HC, can be used as a surrogate marker for abdominal, visceral, and subcutaneous fat estimation, especially in a clinical setting for the young adult population. This study also further established the relationship between abdominal adipose tissue compartments level using MRI, with anthropometric and laboratory measurements in young adulthood.

## 5. Conclusions

This study has shown that MR images, obtained from 1.5 Tesla MRI, can be utilized to assess the abdominal adipose tissue compartments in the young adult population and allow for the semi-automatic identification and quantification of visceral and subcutaneous tissue non-invasively, rather than by examinations performed in clinical imaging diagnoses, such as X-ray examination and CT examination since both expose the human body to harmful radiation. Additionally, a fat assessment from MRI can determine the fat percentage accumulated in each organ. This study also provides a valuable reference for the basic study of the correlation between abnormal metabolism of adipose tissue in different parts of the body and obesity, as well as the occurrence and development of a metabolic syndrome. Interestingly, this study has highlighted the use of simple tools such as anthropometric measurements, especially BMI, to be highly associated with abdominal fat compartments such as subcutaneous fat and abdominal fat. This method could be developed into the primary tool to estimate abdominal fat compartments or could be used to target therapeutic interventions for the obese in young adulthood.

## Figures and Tables

**Table 1:** Demographics, abdominal fat compartments, anthropometric, and laboratory characteristics of young adults in this study.

N = 60	Mean ± SD		
Male/Female (27/33)	Male (27)	Female (33)	*p*-Value
AbdFat%	33.91 ± 12.25	27.99 ± 12.72	0.073
ScFat%	22.48 ± 9.63	21.80 ± 9.99	0.791
VisFat%	10.63 ± 4.95	5.42 ± 3.41	<0.001 *
Age (year)	22.74 ± 2.10	22.21 ± 1.05	0.211
BMI (kg/m^2^)	28.03 ± 4.45	24.04 ± 5.65	<0.05 *
WC (cm)	93.13 ± 14.96	82.51 ± 14.59	<0.05 *
HC (cm)	102.96 ±11.69	97.41 ± 12.55	0.084
W/H ratio	0.90 ± 0.08	0.84 ± 0.06	<0.05 *
FG (mg/dL)	89.19 ± 5.81	85.91 ± 7.92	0.079
Cho (mg/dL)	200.96 ± 40.99	194.27 ± 44.13	0.549
Tri (mg/dL)	114.00 ± 56.62	84.73 ± 35.25	<0.05 *
HDL (mg/dL)	47.26 ± 9.02	57.73 ± 15.71	<0.05 *
LDL (mg/dL)	131.00 ± 35.27	118.57 ± 35.79	0.183
HbA1c (%)	5.26 ± 0.44	5.29 ± 0.68	0.817

AbdFat% = abdominal fat percentage, ScFat% = subcutaneous fat percentage, VisFat% = visceral fat percentages, BMI = body mass index, WC = waist circumference, HC = hip circumference, W/H = waist hip ratio, FG = fasting glucose, Cho = cholesterol, Tri = triglyceride, HDL = high-density lipoprotein, LDL = low-density lipoprotein, HbA1c, Data expressed as mean ± SD, * *p* < 0.05.

**Table 2:** The relationship between demographics, abdominal fat compartments, anthropometric, and laboratory characteristics was performed using partial correlation analysis adjusted for age and sex.

N = 60	Partial Correlation					
Gender (Male/Female) 27/33	Abdominal Fat (%)		Subcutaneous Fat (%)		Visceral Fat (%)	
	R	*p*-Value	r	*p*-Value	r	*p*-Value
BMI (kg/m^2^)	0.824	<0.001 *	0.768	<0.001 *	0.633	<0.001 *
WC (cm)	0.737	<0.001 *	0.707	<0.001 *	0.512	<0.001 *
HC (cm)	0.680	<0.001 *	0.656	<0.001 *	0.467	<0.001 *
W/H ratio	0.547	<0.001 *	0.508	<0.001 *	0.416	0.001 *
FG (mg/dL)	0.369	0.004 *	0.386	0.003 *	0.263	0.048 *
Cho (mg/dL)	0.175	0.190	0.151	0.259	0.165	0.216
Tri (mg/dL)	0.343	0.008 *	0.292	0.026 *	0.352	0.007 *
HDL (mg/dL)	–0.270	0.040 *	–0.227	0.086	–0.261	0.048 *
LDL (mg/dL)	0.247	0.062	0.212	0.109	0.257	0.048 *
HbA1c (%)	0.381	0.003 *	0.351	0.007 *	0.315	0.016 *

* = significant correlation, - = negative correlation, BMI = Body mass index, Cho = cholesterol, FG = fasting glucose, HbA1c = glycated hemoglobin, HC = hip circumference, HDL = high-density lipoprotein, LDL = low-density lipoprotein, Tri = triglyceride, W/H = waist hip ratio, WC = waist circumference, * *p* < 0.05.
